# Aortic waveform responses to insulin in late versus early chronotype with metabolic syndrome

**DOI:** 10.14814/phy2.15473

**Published:** 2022-10-27

**Authors:** Mary‐Margaret E. Remchak, Emily M. Heiston, Anna Ballantyne, Brielle L. Dotson, Steven K. Malin

**Affiliations:** ^1^ Rutgers University New Brunswick New Jersey USA; ^2^ University of Virginia Charlottesville Virginia USA; ^3^ Virginia Commonwealth University Richmond Virginia USA; ^4^ Division of Endocrinology, Metabolism & Nutrition Rutgers University New Brunswick New Jersey USA; ^5^ New Jersey Institute for Food, Nutrition and Health Rutgers University New Brunswick New Jersey USA; ^6^ Institute of Translational Medicine and Science Rutgers University New Brunswick New Jersey USA

**Keywords:** augmentation index, insulin resistance, obesity and inflammation, pulse pressure, pulse wave analysis

## Abstract

Late chronotype (LC) correlates with reduced metabolic insulin sensitivity and cardiovascular disease. It is unclear if insulin action on aortic waveforms and inflammation is altered in LC versus early chronotype (EC). Adults with metabolic syndrome (*n* = 39, MetS) were classified as either EC (Morning‐Eveningness Questionnaire [MEQ] = 63.5 ± 1.2) or LC (MEQ = 45.5 ± 1.3). A 120 min euglycemic clamp (40 mU/m^2^/min, 90 mg/dL) with indirect calorimetry was used to determine metabolic insulin sensitivity (glucose infusion rate [GIR]) and nonoxidative glucose disposal (NOGD). Aortic waveforms via applanation tonometry and inflammation by blood biochemistries were assessed at 0 and 120 min of the clamp. LC had higher fat‐free mass and lower VO_2_max, GIR, and NOGD (between groups, all *p* ≤ 0.05) than EC. Despite no difference in 0 min waveforms, both groups had insulin‐stimulated elevations in pulse pressure amplification with reduced AIx75 and augmentation pressure (AP; time effect, *p* ≤ 0.05). However, EC had decreased forward pressure (Pf; interaction effect, *p* = 0.007) with insulin versus rises in LC. Although LC had higher tumor necrosis factor‐α (TNF‐α; group effect, *p* ≤ 0.01) than EC, both LC and EC had insulin‐stimulated increases in TNF‐α and decreases in hs‐CRP (time effect, both *p* ≤ 0.01). Higher MEQ scores related to greater insulin‐stimulated reductions in AP (*r* = −0.42, *p* = 0.016) and Pf (*r* = −0.41, *p* = 0.02). VO_2_max correlated with insulin‐mediated reductions in AIx75 (*r* = −0.56, *p* < 0.01) and AP (*r* = −0.49, *p* < 0.01). NOGD related to decreased AP (*r* = −0.44, *p* = 0.03) and Pf (*r* = −0.43, *p* = 0.04) during insulin infusion. LC was depicted by blunted forward pressure waveform responses to insulin and higher TNF‐α in MetS. More work is needed to assess endothelial function across chronotypes.

## Graphical abstract

Self‐identified later chronotype adults with metabolic syndrome have blunted aortic waveform responses, and greater inflammation with insulin stimulation compared with early counterparts. Additionally, decreased aerobic fitness and impaired nonoxidative glucose disposal were both associated with aortic waveform responses to insulin. Future work should consider assessing the association of aortic waveform responses to insulin relative to aerobic fitness and metabolic function in later chronotype in the hopes of addressing cardiovascular disease risk prevalent within this classification.

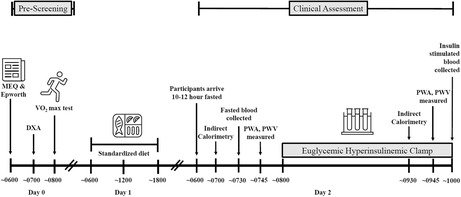

## 1 INTRODUCTION

Metabolic syndrome (MetS) is defined as having three or more risk factors including central obesity, high triglycerides (TG), low high‐density lipoprotein cholesterol (HDL‐c), elevated blood pressure (BP), and/or hyperglycemia (Swarup et al., 2021). In the United States, the prevalence of developing MetS is 24% in men and 22% in women, thereby placing many people at risk for cardiovascular disease (CVD; Swarup et al., 2021). Although the exact pathophysiology of MetS remains to be elucidated, endothelial dysfunction, reduced insulin sensitivity, and inflammation are important (Abdul‐Ghani et al., 2019; Einarson et al., 2018; Swarup et al., 2021). Emerging evidence also suggests that impaired or misaligned circadian rhythms may accentuate these underlying pathophysiological factors in MetS and promote CVD (Chasens et al., 2021). To date, however, limited work has been performed in understanding how chronobiology may impact CVD risk.

People who are characterized as late chronotype (LC; i.e., preference to wake up late and/or perform more activity later in the day) are more prone to disruption in circadian rhythms compared with individuals identifying as early chronotype (EC). This is, in part, due to adjustments to social and/or work schedules. Indeed, LCs tend to have a greater prevalence of MetS than EC, including CVD‐related risk factors such as high TG and CRP, with low HDL‐c and decreased physical activity (Cabrera et al., 2021). Previous work has also suggested that in healthy populations, chronotype influences the diurnal variation of endothelial vasodilation when assessed via flow‐mediated dilation (FMD; Facer‐Childs et al., 2019). Specifically, LC may increase one's risk of having a cardiac event during the evening (i.e., myocardial infarction, stroke; Papaioannou et al., 2006). These findings align with increased intensity of reflected waves occurring in the morning with elevated BP and heart rate (HR) that precede cardiac events, and an additional second peak occurring mid‐to‐late evening (Papaioannou et al., 2006). More recent work also highlights that LC may have social jet lag (i.e., change in wake/sleep schedule between weekdays to weekends; Chellappa et al., 2019), which places them at higher risk for cardiac events. This circadian misalignment effect of increased waketime BP and decreased vagal modulation on cardiac events has been associated with increased inflammation (i.e., hs‐CRP, TNF‐α, IL‐6, resistin; Chellappa et al., 2019), although no work has examined this across chronotypes. Recently, we demonstrated that insulin reduces aortic waveform components (i.e., augmentation index corrected to the HR of 75 beats/min [AIx75], augmentation pressure [AP], forward wave component [Pf]; backward wave component [Pb]) and increases pulse pressure amplification (PPA) in people with MetS (Dotson et al., 2021). Although these data confirm observations that insulin is a vasodilatory hormone that contributes to increasing peripheral blood flow and arterial diameter within the arterial tree (Gordin et al., 2019), it remains unknown if insulin has the similar vascular effects between chronotypes. This is warranted since we recently reported work that LCs have lower metabolic insulin sensitivity and altered fuel selection as supported by TCA cycle intermediate differences compared with EC (Remchak et al., 2022). As such, we sought to fill this vasculature knowledge gap and test the hypothesis that individuals with MetS who identify as LC would present with blunted declines in aortic waveforms in response to insulin via the euglycemic hyperinsulinemic clamp compared with EC. Additionally, we hypothesized these blunted aortic waveform responses in LC may relate to reduced metabolic insulin sensitivity as well as elevated inflammation.

## 2 MATERIALS AND METHODS

### 2.1 Study design and participants

**FIGURE 1:**
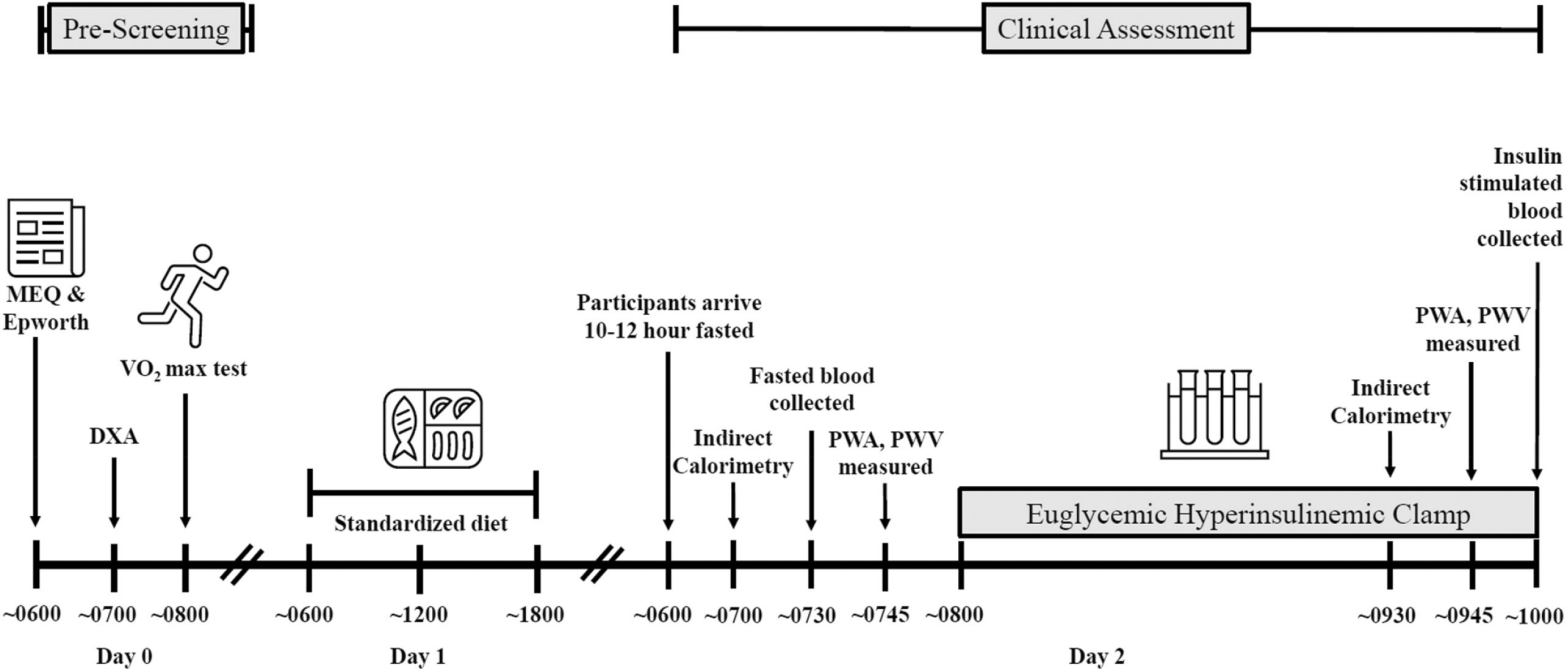
A conceptual timeline of prescreening and clinical assessments. Participants participated in a prescreening assessment that included the collection of aerobic fitness and body composition. A standardized diet was provided 24 h prior to participants' arrival at the clinical research unit for clinical assessment and included a full day's meal. Fasted and insulin‐stimulated measures were collected at 0 and 120 min of the euglycemic hyperinsulinemic clamp.

Thirty‐nine sedentary individuals with MetS according to ATP III criteria (Huang, 2009) were recruited for this cross‐sectional study via social media and/or newspaper flyers from the Charlottesville, VA community. Participants were rank ordered and classified using the Morningness‐Eveningness Questionnaire (MEQ) as either early or late per MEQ scores. Specifically, people with scores 59–86 we considered EC (i.e., definite and/or moderate morning types), whereas LC reflects individuals with scores 31–58 (i.e., intermediate and moderate evening types) to test if chronotype impacts central hemodynamic responses to insulin. Some data (*n* = 34) were previously reported (Remchak et al., 2022) but are shown herein given relevance to interpretations. The Epworth Sleepiness Scale was also used to assess the likelihood of nodding off or falling asleep during specific daily activities (i.e., watching tv, sitting inactive, in a car while stopped, etc.) as previously described (Crook et al., 2019). Individuals between the ages of 40–70 years were included if nonsmoking, physically inactive (exercise <60 min/week, ≤1.5 METs expended per activity; Katzmarzyk et al., 2018), and weight stable (<2 kg weight change during last 3 months). Prior to clinical testing, individuals underwent a physical exam with blood/urine chemistries and resting to maximal stress test electrocardiogram to rule out disease diagnosis (i.e., renal, hepatic, T2D, cardiovascular, etc.) and medication or dietary supplement use (e.g., metformin, SGLT‐2 inhibitors, fish oils, etc.). Participants were excluded from the study if currently taking medications known to impact insulin sensitivity (e.g., biguanides, GLP‐1 agonists, etc.) or improve vasodilation (e.g., Ca^++^ channel blockers, α‐blockers, β‐blockers, nitrates, etc.). Female participants were asked to indicate the status of menses and were noted if currently on oral contraceptives or use of hormone replacement treatment. Participants were provided a conceptual timeline (Figure 1) as well as written and verbal informed consent before participation, as approved by the Institutional Review Board. This study is part of a larger Clinical Trial (Registration # NCT03355469).

### 2.2 Body composition and cardiorespiratory fitness

Total body weight was assessed on a digital scale measured to the nearest 0.10 kg with participants wearing minimal clothing and without shoes. Height was assessed with a stadiometer measured to the nearest 0.10 cm. Body mass index (BMI) was then calculated. Body fat and fat‐free mass (FFM) were measured using dual‐energy X‐ray absorptiometry (Horizon DXA System) following an appropriate 4 h fast of food and beverage, including water, and abstaining a minimum of 4 h from engaging in physical exercise. Waist circumference (WC) was collected 2 cm above the umbilicus using a tape measure to the nearest 0.10 cm twice and averaged. Participants completed a continuous incremental treadmill exercise test with indirect calorimetry to assess maximal oxygen consumption (VO_2_max) using standard criteria, as described before by our lab (Dotson et al., 2021).

### 2.3 Metabolic control

Participants were requested to refrain from the consumption of alcohol, caffeine, medications, and engagement in strenuous physical activity 24 h prior to the study visits. A low‐fat American Heart Association‐based diet consisting of 55% carbohydrates, 15% protein, and 30% fat, with <10% from saturated fat, was provided as a standardized diet. Diets were provided 24 h prior to clamp assessments and included breakfast, lunch, dinner, and two snacks. Energy requirements were determined via fasted resting metabolic rate (RMR) from indirect calorimetry multiplied by a physical activity factor of 1.2 (Harris & Benedict, 1918).

### 2.4 Euglycemic hyperinsulinemic clamp

Participants arrived at the Clinical Research Unit between 06:00 and 08:00 after an approximate 10–12 h overnight fast. A catheter was placed in the antecubital as well as the dorsal hand or forearm vein for infusion and blood sampling, respectively. A primed (250 mU/m^2^/min) constant infusion (40 mU/m^2^/min) of insulin was administered via peristaltic infusion pumps for 120 min. A variable infusion of glucose was also administered to maintain plasma glucose of 90 mg/dL, and plasma glucose was collected every 5 min to determine the appropriate glucose infusion rate (GIR). Blood was obtained prior to the start of insulin infusion (0 min; baseline) and at 90, 105, and 120 min to assess insulin concentrations, which was averaged to define steady state insulin. Inflammation was also determined at 0 and 120 min and included: tumor necrosis factor (TNF‐α), high‐sensitivity C‐reactive protein (hs‐CRP), vascular cell adhesion molecule 1 (VCAM‐1), intercellular adhesion molecule 1 (ICAM‐1), as well as matrix metalloproteinase‐1 (MMP‐1) and matrix metalloproteinase‐7 (MMP‐7). Metabolic insulin sensitivity was defined as the GIR during the last 30 min of the clamp. Although it is assumed that 100% of glucose infused is metabolized, we calculated nonoxidative glucose disposal (NOGD) via indirect calorimetry (GIR—total carbohydrate oxidation) to discern the fate of glucose metabolism.

### 2.5 Pulse waveform analysis

Aortic waveform and hemodynamic measurements were recorded at 0 and 120 min of the clamp using applanation tonometry via the SphygmoCor XCEL system (AtCor Medical). Measurements were collected with participants resting semi‐supine in a temperature‐controlled room (range: 22–23°C). A blood pressure cuff was placed on the participant's upper left arm opposite catheter placement, and three measures were recorded. Data were averaged to provide an augmentation index corrected for a standardized HR of 75 beats per min (bpm; AIx75), AP, brachial systolic (bSBP) and diastolic (bDBP) blood pressure, HR, central systolic (cSBP) and diastolic (cDBP) blood pressure, brachial (bPP) and central (cPP) pulse pressure, and mean arterial pressure (MAP). Central forward pressure (Pf), backward pressure (Pb), and wave reflection magnitude (Pb/Pf × 100; Westerhof et al., 2006) were characterized through deconvolution analysis. PPA was calculated as a ratio (bPP/cPP; Benetos et al., 2010).

### 2.6 Biochemical analysis

Plasma glucose was analyzed by a glucose oxidase assay (YSI Instruments 2700). Other samples were centrifuged at 4°C for 10 min at 1500 *g* and stored at −80°C until later analysis. ELISA analyses were performed to assess plasma insulin (Millipore) as well as TNF‐α, hs‐CRP, VCAM‐1, ICAM‐1, MMP‐1, and MMP‐7 (R&D Systems, INC). Biomarkers were batch analyzed in duplicate to minimize variance within conditions using ELISA.

### 2.7 Statistical analysis

Data were analyzed using SPSS (IBM, V. 28.0). Normality of data was assessed using the interquartile range. Nonnormally distributed data were log‐transformed for analysis. Baseline demographics were compared with independent, two‐tailed Student's unpaired *t* tests. Two‐way (group × time) repeated measures ANOVAs were utilized to understand insulin‐stimulated effects on outcomes between chronotypes as previously done. VO_2_max, body composition, and insulin sensitivity were used as covariates in ANOVA analysis against waveform and inflammation, given these outcomes either differed between groups or are considered important factors in the modification of vascular function. Cohen's *d* effect size was also calculated to assess physiological relevance among group outcomes. Relevance was interpreted as small *d* = 0.2, medium *d* = 0.5, or large *d* = 0.8, respectively. Pearson correlations were used to assess relationships. Data are presented as mean ± SEM, and significance was denoted as *p* ≤ 0.05.

## 3 RESULTS

### 3.1 Participant demographics

There were no significant differences observed between EC and LC with age and body weight (Table 1). Likewise, no differences were observed either in perceived daytime sleepiness, ATP III score, insulin concentrations, or associated cardiometabolic risk factors (Table 1). EC did, however, have higher VO_2_max (*p* = 0.05) and less FFM (*p* = 0.04), as well as greater metabolic insulin sensitivity (GIR, *p* = 0.002) and NOGD (*p* = 0.003, Table 1) versus LC.

**TABLE 1:** Participant demographics

	Early	Late	*p*‐value	Cohen's *d* effect size
Characteristics				
*N* (M/F)	20 (4 M/16F)	19 (3 M/16F)		
Age (years)	55.1 ± 1.2	54.8 ± 1.9	0.93	0.03
Race/ethnicity				
African American	0	2		
Hispanic/Latino	1	0		
White	19	17		
MEQ Score	63.5 ± 1.2	45.5 ± 1.3	<0.001	3.22
Epworth Sleep Score	6.3 ± 0.7	7.4 ± 1.2	0.41	0.27
Body composition				
Weight (kg)	102.2 ± 3.7	105.0 ± 4.5	0.63	0.04
BMI (kg/m^2^)	35.8 ± 1.0	36.9 ± 1.2	0.49	0.23
Fat mass (kg)	43.7 ± 2.7	44.9 ± 1.7	0.70	0.24
FFM (kg)	53.1 ± 0.8	56.5 ± 1.3	0.04	0.86
Body fat (%)	47.0 ± 1.3	44.2 ± 0.9	0.08	0.60
Aerobic fitness				
VO_2_max (ml/kg/min)	23.3 ± 0.8	21.0 ± 0.8	0.05	0.65
VO_2_max (ml/FFM‐kg/min)	42.9 ± 1.8	38.2 ± 1.5	0.05	0.72
Cardiometabolic disease risk				
ATP III Score	3.5 ± 0.1	3.5 ± 0.2	0.91	0.04
WC (cm)	112.3 ± 2.2	114.1 ± 2.6	0.60	0.17
SBP (mmHg)	132.2 ± 3.1	133.6 ± 1.7	0.69	0.13
DBP (mmHg)	78.7 ± 2.2	76.3 ± 1.9	0.42	0.26
FBG (mmol/L)	5.9 ± 0.1	5.7 ± 0.1	0.55	0.17
TG (mmol/L)	1.7 ± 0.3	1.7 ± 0.1	0.89	0.11
HDL‐c (mmol/L)	1.2 ± 0.1	1.3 ± 0.1	0.25	0.47
GIR (mg/FFM‐kg/min)	5.0 ± 0.5	3.1 ± 0.1	0.002	1.21
Fasting Insulin (uU/ml)	16.0 ± 2.3	21.7 ± 2.6	0.11	0.55
Steady state insulin (uU/ml)	87.6 ± 5.3	82.0 ± 5.8	0.48	0.27
NOGD (mg/FFM‐kg/min)	2.7 ± 0.4	1.2 ± 0.2	0.003	1.24

*Note*: Data are presented as mean ± SEM. An independent sample *t* test was utilized to determine group differences between early and late chronotypes. Sample size and sex distribution between groups reported in table (EC: *n* = 20 [4 M/16F]; LC: 19 [3 M/16F]). The effect size was calculated via Cohen's *d*.

Abbreviations: ATP III Score, adult treatment panel III quantified risk of metabolic syndrome; BMI, body mass index; DBP, diastolic blood pressure; FBG, fasting blood glucose; GIR, glucose infusion rate determined as average glucose of the last 30 min of the euglycemic hyperinsulinemic clamp; HDL‐c, high‐density lipoprotein; MEQ Score, Morningness‐Eveningness questionnaire score; NOGD, nonoxidative glucose disposal determined as GIR—total carbohydrate oxidation; SBP, systolic blood pressure; TG, triglycerides; VO_2_max, aerobic capacity relative to mean body weight (kg) and fat‐free mass (FFM); WC, waist circumference.

### 3.2 Central hemodynamics and aortic waveforms

**FIGURE 2:**
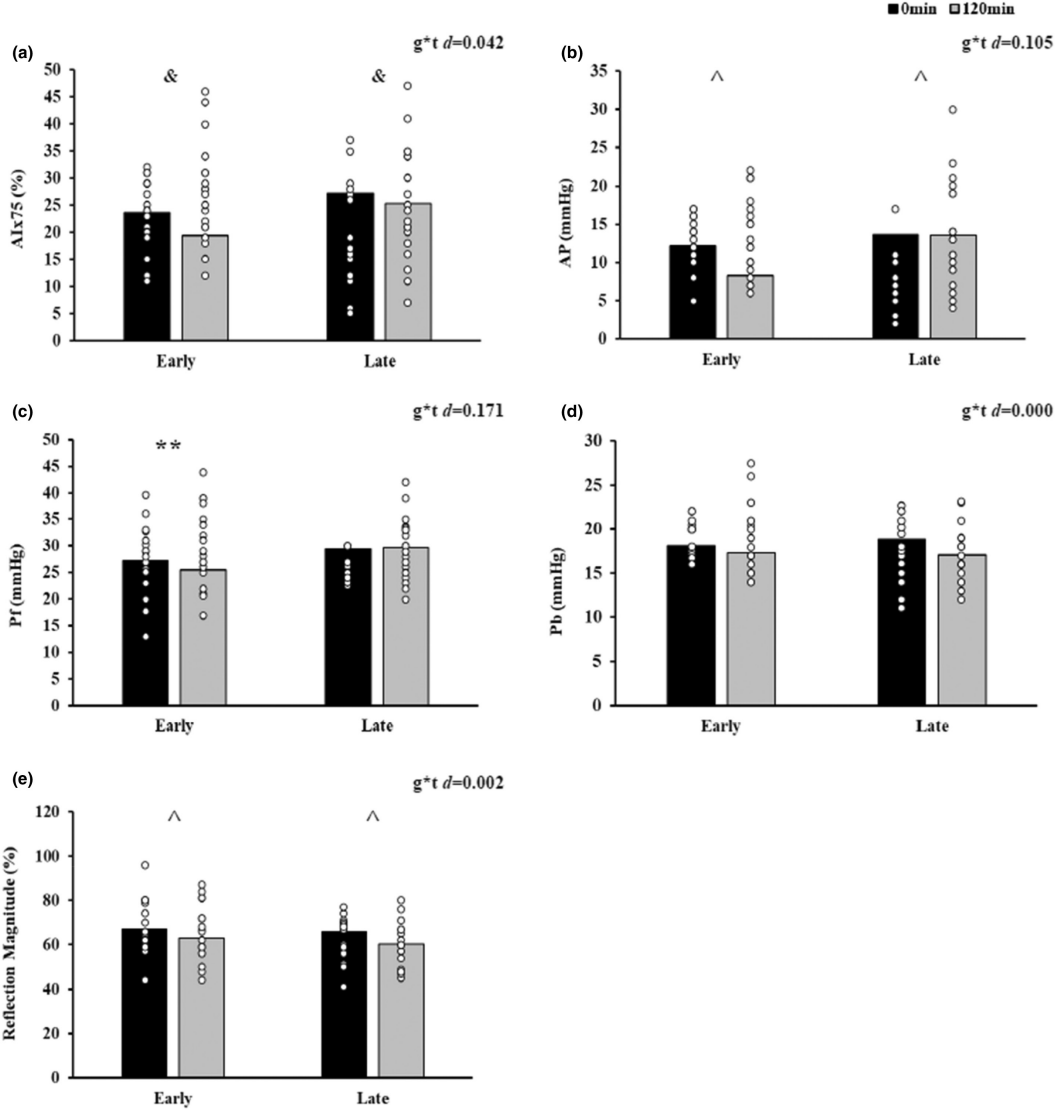
Waveform component analysis. Data are presented as mean ± SEM. Two‐way (group × time) ANOVAs were utilized to assess the influence of insulin stimulation. Aortic waveform reflections at baseline and with insulin stimulation: (a) augmentation index (AIx75; EC: *n* = 19 [4 M/15F]; LC: *n* = 19 [3 M/16F]), (b) augmentation pressure (AP; EC: *n* = 19 [4 M/15F]; LC: *n* = 19 [3 M/16F]), (c) forward wave reflection (Pf; EC: *n* = 17 [3 M/14F]; LC: *n* = 19 [3 M/16F]), (d) backward wave reflection (Pb; EC: *n* = 18 [4 M/14F]; LC: *n* = 19 [3 M/16F]), and (e) reflection magnitude (EC: *n* = 19 [4 M/15F]; LC: *n* = 19 [3 M/16F]). Significant time effect denoted as ^&^ *p* ≤ 0.05 and ^^^ *p* ≤ 0.01. Significant group × time effect denoted as **p ≤ 0.01. The effect size was calculated via Cohen's *d*.

No differences in brachial or central blood pressure during fast or insulin stimulation existed between chronotypes. However, insulin increased HR (time effect, *p* = 0.02) and PPA in both EC and LC (time effect, *p* < 0.001, Table 2). While LC did not differ from EC in fasting aortic waveforms, insulin lowered AIx75 (time effect, *p* ≤ 0.05, Figure 2a) and AP (time effect, *p* ≤ 0.01, Figure 2b) in both chronotypes. Additionally, EC had a lower Pf after insulin stimulation compared with LC (interaction effect, *p* = 0.007, *d* = 0.17, Figure 2c). Although no change was observed in Pb in either group, insulin similarly decreased the reflection magnitude (time effect, *p* < 0.001, Figure 2d,e).

**TABLE 2:** Effect of insulin on blood pressures (BPs) over time in chronotype

	Early	Late	Group effect *p* value	Time effect *p* value	Group × time effect *p* value	Cohen's *d* effect size
Brachial						
Heart rate (bpm)						
0 min	61.5 ± 1.3	64.5 ± 1.9	0.30	0.02	0.98	0.03
120 min	65.8 ± 1.3	67.3 ± 1.9				
Systolic BP (mmHg)						
0 min	132.8 ± 4.9	131.4 ± 2.9	0.98	0.82	0.46	0.00
120 min	131.0 ± 2.6	132.3 ± 2.8				
Diastolic BP (mmHg)						
0 min	81.7 ± 2.7	78.5 ± 2.0	0.46	0.60	0.32	0.17
120 min	79.9 ± 1.9	79.1 ± 2.0				
Pulse pressure (mmHg)						
0 min	52.8 ± 2.8	51.1 ± 2.2	0.37	0.90	0.87	0.00
120 min	51.1 ± 1.7	53.2 ± 2.6				
Central						
Systolic BP (mmHg)						
0 min	122.5 ± 4.3	120.7 ± 2.7	0.93	0.32	0.35	0.04
120 min	119.4 ± 2.4	120.6 ± 2.7				
Diastolic BP (mmHg)						
0 min	82.3 ± 4.3	79.4 ± 2.0	0.39	0.86	0.36	0.04
120 min	81.6 ± 1.9	80.2 ± 2.1				
Pulse pressure (mmHg)						
0 min	39.6 ± 3.8	41.4 ± 1.9	0.23	0.23	0.72	0.06
120 min	37.7 ± 1.7	40.4 ± 2.2				
Pulse pressure amplification						
0 min	1.29 ± 0.05	1.28 ± 0.02	0.61	<0.001	0.62	0.02
120 min	1.32 ± 0.02	1.34 ± 0.02				
Mean arterial pressure (mmHg)						
0 min	96.1 ± 3.5	94.2 ± 2.1	0.69	0.82	0.33	0.03
120 min	94.9 ± 2.0	94.9 ± 2.0				

*Note*: Data are presented as mean ± SEM. Two‐way (group × time) ANOVAs were utilized. Sample size and sex distribution includes brachial measures (EC: *n* = 19 [4 M/15F]; LC: *n* = 19 [3 M/16F]), central measures (EC: *n* = 18 [4 M/14F]; LC: *n* = 19 [3 M/16F]), pulse pressure amplification (EC: *n* = 18 [4 M/14F]; LC: *n* = 19 [3 M/16F]), and mean arterial pressure (EC: *n* = 18 [4 M/14F]; LC: *n* = 19 [3 M/16F]). The effect size was calculated via Cohen's *d*.

### 3.3 Inflammation

**FIGURE 3:**
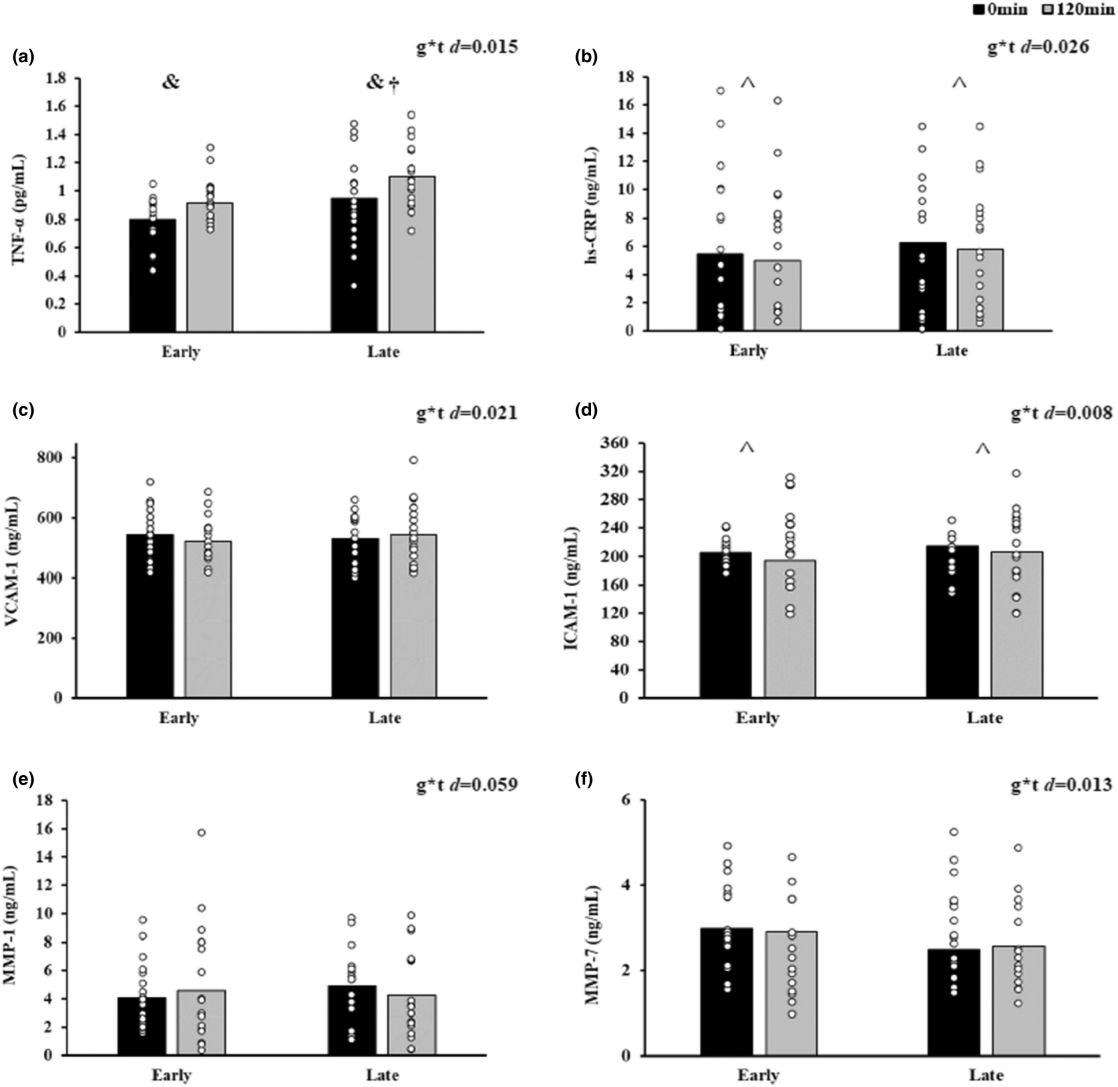
Inflammation and insulin stimulation. Data are presented as mean ± SEM. Two‐way (group × time) ANOVAs were utilized to assess the influence of insulin stimulation across time. Inflammation measures at baseline and with insulin stimulation: (a) tumor necrosis factor (TNF‐α; EC: *n* = 19 [3 M/16F]; LC: 18 [3 M/15F]), (b) high‐sensitivity C‐reactive protein (hs‐CRP; EC: *n* = 20 [4 M/16F]; LC: 17 (3 M/14F)), (c) vascular cell adhesion molecule 1 (VCAM‐1; EC: *n* = 20 [4 M/16F]; LC: 19 [3 M/16F]), (d) intercellular adhesion molecule 1 (ICAM‐1; EC: *n* = 20 [4 M/16F]; LC: 19 [3 M/16F]), (e) matrix metalloproteinase‐1 (MMP‐1; EC: *n* = 20 [4 M/16F]; LC: 18 [3 M/15F]), and (f) matrix metalloproteinase‐7 (MMP‐7; EC: *n* = 18 [4 M/14F]; LC: 16 [2 M/14F]). Significant group effect denoted as ^†^ *p* ≤ 0.01. Significant time effect denoted as ^&^ *p* ≤ 0.05 and ^^^ *p* ≤ 0.01. The effect size was calculated via Cohen's *d*.

Chronotypes did not differ in fasting inflammation. However, TNF‐α increased in response to insulin in both EC and LC (time effect, *p* = 0.03, Figure 3a), although LC had higher TNF‐α overall (group effect, *p* ≤ 0.01). Furthermore, insulin reduced hs‐CRP (time effect *p* ≤ 0.01, Figure 3b) and ICAM‐1 (time effect, *p* ≤ 0.01, Figure 3d). There were no differences in VCAM‐1 (Figure 3c), MMP‐1 (Figure 3e), or MMP‐7 (Figure 3f) responses to insulin for either chronotype.

### 3.4 Correlations

**FIGURE 4:**
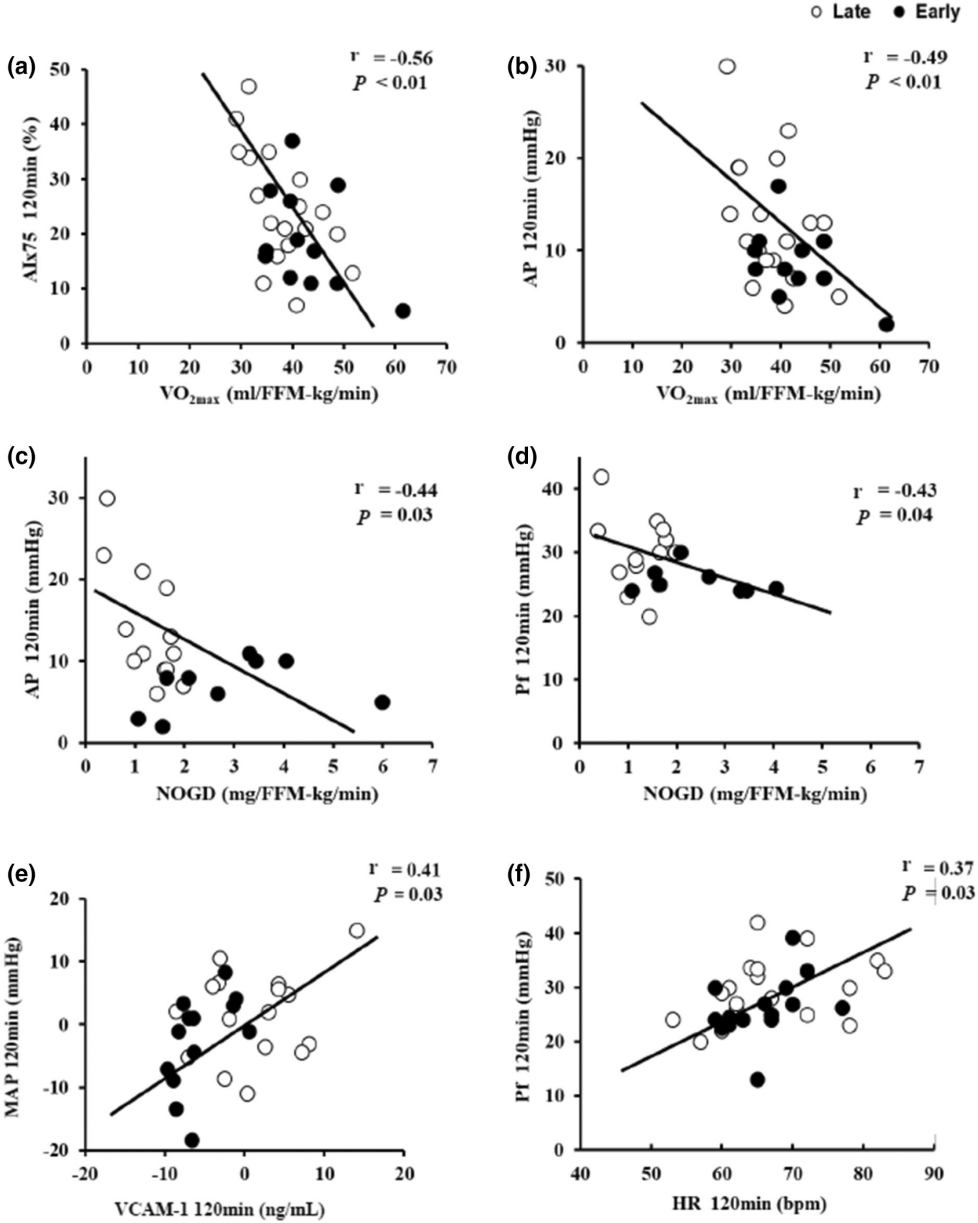
Correlations of insulin‐stimulated aortic waveform components of augmentation index (AIx75), augmentation pressure (AP), forward wave reflection (Pf), and mean arterial pressure (MAP) between aerobic fitness (VO_2max_), clamp‐derived nonoxidative glucose disposal (NOGD), insulin‐mediated change in inflammation, and insulin‐stimulated heart rate (HR). (a) AIx75 120 min versus VO_2max_ (*n* = 35 [7 M/28F]), (b) AP 120 min versus VO_2max_ (*n* = 35 [7 M/28F]), (c) AP 120 min versus NOGD (n = 35 [6 M/29F]), (d) Pf 120 min versus NOGD (*n* = 35 [7 M/28F]), (e) ∆MAP versus ∆VCAM‐1 (*n* = 37 [7 M/30F]), and (f) Pf 120 min versus HR 120 min (*n* = 38 [7 M/31F]).

Fasting indices of aortic waveform components did not correlate with fitness, body composition, or insulin sensitivity (data not shown). However, higher MEQ scores were correlated with lower fasting bPP (*r* = −0.33, *p* = 0.05), cPP (*r* = −0.41, *p* = 0.02), and AIx75 (*r* = −0.36, *p* = 0.03). Further, higher MEQ scores were inversely related to insulin‐stimulated AP (*r* = −0.42, *p* = 0.016) and Pf (*r* = −0.41, *p* = 0.02). VO_2_max significantly correlated with insulin‐stimulated AIx75 (*r* = −0.56, *p* < 0.01, Figure 4a) and AP at 120 min, respectively (*r* = −0.49, *p* < 0.01, Figure 4b). Although metabolic insulin sensitivity (GIR) did not significantly correlate with vascular measures of AIx75 (*r* = −0.19, *p* = 0.36), AP (*r* = −0.24, *p* = 27), or Pf (*r* = −0.24, *p* = 0.29) during the clamp, NOGD related to AP (*r* = −0.44, *p* = 0.03, Figure 4c) and Pf at 120 min, respectively (*r* = −0.43, *p* = 0.04, Figure 4d). Insulin‐mediated VCAM‐1 also correlated with lower MAP during the clamp (*r* = 0.41, *p* = 0.03, Figure 4e). Elevations in HR following insulin infusion also correlated with increases in Pf at 120 min (*r* = 0.37, *p* = 0.03, Figure 4f).

## 4 DISCUSSION

LC has been suggested to raise CVD risk in part through dyslipidemia and/or hyperglycemia (Cabrera et al., 2021; Reutrakul et al., 2013). However, individuals in the current study had similar levels of TG, HDL, and glucose as well as MetS ATP III scores. This suggests that factors other than altered substrates can contribute to CVD risk in LC. The main findings of the current study demonstrate that LC had a rise in Pf during the clamp compared with EC, despite insulin lowering AIx75 and AP in both EC and LC. However, higher MEQ scores (i.e., EC) were associated with greater reductions in AP and Pf during insulin stimulation. When considering that fasting aortic waveform components are comparable in EC and LC, these findings support the hypothesis that later chronotypes may have elevated CVD risk due to at least partially blunted insulin‐mediated aortic waveforms (Angoff et al., 2021; Knutson & von Schantz, 2018).

Recent work purports that reduced insulin sensitivity via fasting surrogates might play a role in contributing to this heightened CVD risk (Reutrakul et al., 2013). Indeed, we confirm such observation by highlighting with the clamp that LCs have lower metabolic insulin sensitivity than EC. Given links between metabolic insulin sensitivity and CVD risk (Heiston & Malin, 2019; Pinkney et al., 1997), it was interesting that we observed a relationship between NOGD and aortic waveform components during the clamp in the present work. As a result, these findings highlight potential disturbances in the ability of insulin to promote nonoxidative glucose metabolism in relation to blunted declines in aortic waveforms. Additional work examining chronotype classifications along the arterial tree is warranted to gain insight into how insulin impacts nutrient delivery and utilization per chronotype.

We and others have previously shown that insulin reduces AIx75 and AP during euglycemia in adults with MetS (Dotson et al., 2021) and without metabolic disease (Jahn et al., 2016). Herein, we expand upon this work by showing that chronotype may influence this response. Although it is beyond the scope of the present study to determine how insulin differently acted on aortic waveforms, several potential reasons exist. LC is associated with a less healthy diet, including higher fat intake and more frequent snacking (Cabrera et al., 2021; Kanerva et al., 2012; Sato‐Mito et al., 2011). While this study was not designed to examine appetite regulation, it is important to note that individuals were fed for 24 h prior to the aortic waveform investigation. Thus, the influence of acute dietary intake on respective outcomes should be minimized. In addition, it would not be surprising if LCs have more body fat due to poor dietary habits compared to EC. However, we observed no difference in BMI or WC, suggesting that body fat was similar between chronotypes. In fact, when using DXA, total body fat tended to be higher and FFM was lower in EC compared with LC. As a result, body fat quantity is unlikely to explain these altered effects of insulin on aortic waveform profiles in LC.

Aerobic fitness is another factor associated with attenuated blood pressure components (Donley et al., 2014; Jung et al., 2014; Sugawara et al., 2015). In line with this, LCs have been reported to have lower physical activity levels, which may contribute to CVD risk and arterial stiffness (Blair et al., 1996; Jae et al., 2010). We noted that EC had higher VO_2_max compared with LC, whether scaled to body weight or FFM. This suggests that fitness may have contributed to greater effects of insulin to reduce aortic waveforms in people with EC independent of skeletal muscle mass. Additional studies have demonstrated that prolonged aerobic exercise may promote beneficial cardiovascular adaptations leading to reduced sympathetic drive and arterial stiffness (Kang et al., 2016; Mora‐Rodriguez et al., 2017). Indeed, this claim is supported by the correlation between VO_2_max and insulin‐stimulated AIx75 as well as AP in the present work. This is also consistent with others showcasing that fitness enhances endothelial nitric oxide (NO) bioavailability (Kim et al., 2014; Zhang et al., 2006). Improved NO is an important adaptation as it influences the vascular tone and may improve arterial compliance, which has the capability to protect against age‐related declines in vascular health (Wilkinson et al., 2002, 2004). However, it is important to acknowledge aortic waveform comparisons between chronotype groups were statistically covaried for aerobic fitness. Thus, we interpret these preliminary data as expanding on the notion that while fitness modulates insulin action on the vasculature, chronotype appears to have independent effects on insulin‐stimulated aortic waveform profiles. Additional work is required to confirm this and test how exercise modulates insulin action on aortic waveforms and/or vascular function to optimize cardiometabolic health across chronotypes (Eichner et al., 2019; Gilbertson et al., 2020; Heiston et al., 2021; Sun et al., 2014).

Inflammation is an important modifier of both insulin and aortic waveforms through, in part, reduced NO bioavailability (Janus et al., 2016). In fact, individuals who are considered insulin sensitive have lower inflammatory cytokine levels (Sun et al., 2014) and higher vessel compliance as well as reduced blood pressures (Muniyappa et al., 2007; Zheng & Liu, 2015). This later point is clinically relevant as LC may have a higher hypertension risk than EC (Merikanto et al., 2013), potentially due to inflammation (i.e., ICAM‐1, TNF‐α; Huang et al., 2016). In the present work, we observed higher TNF‐α in LC compared with EC (i.e., group effect). Yet, insulin raised TNF‐α and lowered hs‐CRP in both chronotypes. While there was much variation in response to insulin for the inflammatory markers measured in the present work, reductions in VCAM‐1 during insulin were associated with insulin‐stimulated declines in MAP. This observation, as well as the declines in AP, suggest that reduced inflammation in response to insulin may contribute to improved aortic blood pressure. Interestingly, this is consistent with epidemiological evidence proposing that social jet lag and night shift work may elevate CVD risk via increased levels of hs‐CRP (Leproult et al., 2014) and decreased life expectancy (Tan & Scott, 2014). Furthermore, in conditions of endothelial dysfunction, NO bioavailability is often reduced in relation to increased expression of VCAM‐1 and ICAM‐1 through enhanced reactive oxidative stress in the vessel walls (Wright et al., 2015).

It is worth recognizing that blood pressure was not different between EC or LC during fasting or insulin‐stimulated conditions. In fact, insulin raised PPA and lowered reflection magnitude in both EC and LC in the current study. This suggests that insulin reduced the pressure load on the heart relative to that of the periphery. Interestingly, however, we noted significant associations between elevations in HR with increases in Pf during insulin‐stimulated states. In particular, LC had presented with higher Pf during insulin stimulation than EC despite both groups showing an approximate 3 bpm rise in HR. A potential explanation for these outcomes may be a central compensation mechanism via HR to maintain necessary blood flow to the periphery with insulin stimulation in both groups, although LCs demonstrate an altered left ventricular ejection pattern (Xiao et al., 2018). This altered ejection pattern may be explained as potentially shortened ejection duration propagating a greater Pf and increasing the risk of left ventricular heart failure (Kolev & Zimpfer, 1995; Sharman et al., 2009; Torjesen et al., 2014). In either case, the clinical ramifications of this attenuated decline in AIx75 and AP and rise in Pf seen within LC during insulin stimulation are still somewhat unclear despite our findings being similar to work highlighting that social jetlag or night shift work is related to elevated atherosclerosis risk (Haupt et al., 2008; Kantermann et al., 2013). Because recent work suggests chronic circadian misalignment due to daily adjustments for modern work schedules and sleep cycles may promote increased hs‐CRP and TNF‐α (Wright et al., 2015), additional work on vascular insulin sensitivity with chronotype is needed to optimize targeted treatment plans.

We are mindful that this study has limitations that may impact interpretations. Participants were grouped as either EC or LCs dependent upon the 50th percentile according to previously conducted protocols (Remchak et al., 2022). While this proof of concept enables the determination of chronotype type, we are not able to comment on chronotype subgroups (e.g., definite morning, moderate morning, intermediate, moderate evening, definite evening) due to small sample sizes. As such, we identify our groups under the broad definition of early to include moderate and definite morning (MEQ score ≥ 59; *n* = 18 moderate morning, *n* = 2 definite morning) or late to include intermediate and moderate evening (MEQ score ≤ 58; *n* = 13 intermediate, *n* = 6 moderate evening). Despite this study likely being underpowered for some outcomes given the study design, this is the first study to assess insulin‐stimulated aortic waveforms between chronotypes. These data should be considered preliminary in nature to help establish future prospective studies across chronotype‐specific groups. Further, given our sample size, we are unable to analyze the impact race and/or sex may have on vascular responses to insulin between chronotypes. Menstrual status has been raised as an important modifier of vascular function across age (Shenouda et al., 2018). While we do not have statistical power to discern menstrual status role on the present findings, menstrual status was similar between LC (*n* = 12 postmenopausal, *n* = 1 perimenopausal, *n* = 3 premenopausal) and EC (*n* = 12 postmenopausal, *n* = 1 perimenopausal, *n* = 2 premenopausal) chronotypes. No woman reported using oral contraceptives or hormone replacement therapy. Since both groups were predominantly postmenopausal, menstrual status was unlikely to have impacted the observed chronotype effects. We acknowledge, nonetheless, that more work is needed examining oral contraceptive (Hampson, 2020) and/or hormonal replacement therapy (Agarwal et al., 2018) influence on vascular insulin action (Stanczyk et al., 2013) given the public health relevance. Additionally, sleep duration and quality were not directly recorded prior to the day of the clamp, though we did assess perceived habitual sleep patterns via the Epworth questionnaire and found no significant difference in the degree of doziness between groups. It should also be acknowledged that statin/BP medications may have anti‐inflammatory properties and affect the results of the current study (Quist‐Paulsen, 2010). Medication status relative to dyslipidemia and BP/HR was comparable between LC (*n* = 1 statin, *n* = 7 BP) and EC (*n* = 3 statin, *n* = 5 BP), and all people discontinued medication 24 h prior to clamping procedures to minimize this effect. Individuals were studied under euglycemia with hyperinsulinemia. Whether studied under hyperglycemia (or elevated free fatty acids) with hyperinsulinemia, understanding the extent of the metabolic state modifications of aortic waveforms remains unknown (Horton et al., 2021). Last, pulse wave velocity is considered the gold standard indicator of arterial stiffness and was not assessed here. Despite these limitations (Cheng et al., 2007), we utilized AIx75 as an estimate of aortic stiffness since it is associated with CVD risk (Nürnberger et al., 2002).

In conclusion, adults with MetS characterized as LC have altered aortic waveforms in response to insulin. Additionally, LCs appear to have greater TNF‐α during insulin infusion compared with ECs. Decreased VO_2_max and impaired nonoxidative glucose disposal were associated with aortic waveform responses to insulin. These associations suggest that aortic waveforms responses to insulin are related to aerobic fitness and the ability of insulin to favor glucose disposal. Future work should consider identifying the mechanism(s) that contribute to this reduced vascular insulin sensitivity in LCs in an effort to optimize treatments that prevent or attenuate CVD progression as well as development.

## AUTHOR CONTRIBUTIONS

Steven K. Malin conceptualized the study and methodology. Mary‐Margaret E. Remchak, Emily M. Heiston, Anna Ballantyne, Brielle L. Dotson, and Steven K. Malin contributed to data collection. Mary‐Margaret E. Remchak takes responsibility for primary statistical analysis. Mary‐Margaret E. Remchak and Steven K. Malin co‐wrote the original draft, while Emily M. Heiston, Anna Ballantyne, and Brielle L. Dotson provided edits. All authors have read and agreed to the final published version of the manuscript.

## FUNDING INFORMATION

This work was supported by the National Institutes of Health RO1‐HL130296 (SKM).

## CONFLICT OF INTEREST

The authors report no conflict of interest.

## ETHICS STATEMENT

This study was conducted in accordance with The Declaration of Helsinki (1964), except for registration in database.
